# Genome-Wide Identification and Expression Profiling of the TGA Gene Family in Rapid-Cycling *Brassica rapa*

**DOI:** 10.3390/cimb48101089

**Published:** 2026-10-21

**Authors:** Tongtong Li, Xiaoying Wu, Lei Cheng, Huihui Guo, Fanchang Zeng

**Affiliations:** 1 State Key Laboratory of Crop Biology, College of Agronomy, Shandong Agricultural University, Tai’an 271018, China; lttsdau2019@163.com (T.L.); 18369503515@163.com (X.W.); 2 College of Resources and Environment, Shandong Agricultural University, Tai’an 271018, China; leicheng@sdau.edu.cn

**Keywords:** rapid-cycling *Brassica rapa*, TGA gene family, phylogenetic analysis, expression analysis, protein structure prediction

## Abstract

TGA transcription factors are key regulators within the plant bZIP superfamily, coordinating stress adaptation as well as plant growth and development. Rapid-cycling *Brassica rapa* (RCBr) is a valuable genetic resource for investigating gene functions in *Brassica rapa*; however, systematic investigations of its TGA gene family remain limited. In this study, we performed a genome-wide identification of the TGA gene family and identified 19 *BrTGA* genes in RCBr. Subsequent bioinformatic analyses revealed that the *BrTGA* family exhibited conserved protein sequences but divergent gene structures. Quantitative expression profiling across multiple tissues revealed that most *BrTGA* genes were predominantly expressed in roots, with several members exhibiting distinct tissue-preferential expression patterns. These findings provide valuable insights and genetic resources for further investigating the regulatory functions of TGA transcription factors in *B. rapa*.

## 1. Introduction

Transcriptional regulation governs plant biological processes, and the basic region leucine zipper (bZIP) transcription factor family, one of the largest gene families, is widely distributed in animals, plants and microorganisms [1,2,3]. The TGA gene family, a significant subgroup within the clade D of bZIP factors in *Arabidopsis thaliana*, has garnered extensive attention due to its conserved structure and diverse functions [4]. TGA transcription factors specifically recognize and bind to the TGACG motif (known as activation sequence-1 or as-1) in the promoters of target genes [5]. Typical TGA proteins possess a conserved bZIP domain as well as two glutamine-rich transcriptional-activation motifs at the C-terminus [6,7]. In recent years, TGA family members have been identified across various genomes, with the number of members showing significant variation between species [8,9]. These studies collectively establish the fundamental role of the TGA family in plant physiology and ecological adaptation, making it a persistent focus of interest in the plant molecular biology community [10].

The TGA gene family was first reported to be involved in the regulation of systemic acquired resistance [11]. TGA transcription factors such as tobacco TGA2.2 can bind to the as-1 cis-element and regulate SA-mediated expression of downstream genes [12]. TGA members in sugarcane and papaya have also been confirmed to participate in defense responses [13,14].

TGA transcription factors also exhibit broad and precise regulatory roles in plant growth, development, and environmental adaptation [11,12,13]. The TGA family widely participates in the development of multiple plant organs, including inflorescences [14], floral organs [15,16], and roots [12,17]. SlTGA2.2 (*Solanum lycopersicum*) modulates hormone signaling and maturation-related transcription factors to control fruit growth and ripening [13]. Rice TGA members also regulate key agronomic traits such as tiller angle, further expanding the regulatory roles of TGA genes in plant architecture [18].

Accumulating evidence has revealed that TGA proteins serve as critical molecular integrators linking diverse developmental signaling pathways. In *A. thaliana*, PAN recruits NPR1-like proteins BOP1/2 to the promoters of developmental genes such as *AP1*, thereby modulating floral meristem identity [16,19]. At the same time, TGA7 maintains auxin homeostasis by directly activating the expression of *PILS2*, suggesting an essential role of TGA members in auxin-dependent growth regulation [20]. TGA4/OBF4 interacts with CO to modulate *FT* expression, coordinating floral transition and plant defense responses in *A. thaliana* [21]. In addition to predominant developmental roles, several TGA members also participate in environmental signal adaptation. Clade II TGA2/5/6 contribute to UV-B tolerance by activating GSTU-mediated ROS scavenging [22]. TGA1/4 dynamically regulate nitrate uptake in response to fluctuating nitrogen conditions [23], and rice OsbZIP83 is involved in iron homeostasis [24]. Moreover, TGA factors modulate the expression of detoxification enzymes such as P450, contributing to the natural variation in herbicide tolerance in plants [25].

TGA factors are tightly regulated via post-translational modifications [26]. The Glutaredoxin (GRX) family acts as a crucial regulator of TGA activity. For example, CC-type GRXs physically interact with TGA proteins, such as PAN [27], FEA4 [14], TGA9/10 [28], and MeTGA2 [29]. The redox status of cysteine residues in TGA proteins is affected by this interaction, which further impacts the DNA-binding capacity of TGA proteins, transcriptional activity, and subcellular localization.

To date, genome-wide identification of TGA gene families has been completed in numerous plant species, including *A. thaliana*, *Oryza sativa* (rice), *Ananas comosus* (pineapple), *Glycine max* (soybean) and *Phaseolus vulgaris* (common bean), laying a solid foundation for uncovering their conserved and divergent biological functions. Nevertheless, relevant systematic investigations targeting the TGA gene family in RCBr have not yet been reported. RCBr is a rapid-flowering germplasm artificially bred by the University of Wisconsin-Madison [30]. Such favorable traits render RCBr an ideal model material for exploring the regulatory crosstalk between abiotic stress adaptation and plant growth and development. Accordingly, in this study, we performed comprehensive genome-wide identification and bioinformatic analyses of the TGA family genes in RCBr.

## 2. Materials and Methods

### 2.1. Plant Materials and Growth Conditions

All plant materials were cultured in plant growth rooms with controlled temperature and photoperiods. RCBr materials were maintained at 23 °C under 24 h continuous light.

### 2.2. Identification of TGA Family Members in the Genomes of Two Brassica rapa Accessions, RCBr and Chiifu

In this study, the *Brassica_rapa*_FPsc_v1.3 genome assembly was used as the reference genome for RCBr. To identify TGA family members in the RCBr genome, 10 previously reported AtTGA protein sequences were retrieved from TAIR (https://www.arabidopsis.org/ accessed on 11 June 2026) and used as queries. Since TGA proteins belong to the bZIP Group D subfamily and contain a conserved bZIP domain, candidate TGA genes were identified through a combination of BLASTP homology searches (performed using TBtools-II, v2.487) and conserved domain analysis. BLASTP searches against the RCBr protein database using an E-value threshold ≤ 1 × 10^−5^ initially identified 29 candidate proteins. These candidates were subsequently analyzed using the CDD and Pfam databases for bZIP domain validation, and sequences lacking a complete bZIP domain were excluded, resulting in 20 non-redundant TGA candidates.

To further confirm the classification of BrTGA proteins, an HMM-based search using the bZIP domain profile was performed (E-value ≤ 1 × 10^−5^) (Table S1), followed by multiple sequence alignment and phylogenetic analysis. Based on phylogenetic clustering, 19 candidates were assigned to the bZIP Group D (TGA) subfamily and were designated as *BrTGA* genes. Collectively, 19 *BrTGA* family members were identified through integrated BLASTP, HMM (performed using TBtools-II, v2.487), and phylogenetic analyses.

The identification pipeline and all parameter settings for TGA members in the Chiifu genome were identical to those used for RCBr.

### 2.3. Phylogenetic Analysis and Protein Characterization of BrTGA Family Members

The lists of TGA gene family members in *A. thaliana*, *Brassica napus* L., and *O. sativa* were obtained from previously published literature [7,11,31], and those in *Brassica rapa* subsp. *pekinensis* cv. Chiifu were identified following the same procedure as described earlier for *BrTGA* genes. Homologous TGA sequences of these species were retrieved to construct a phylogenetic tree using the one-step ML tree function in TBtools (v2.487) [32], with 5000 bootstrap replicates adopted as the default setting of the program, with visualization optimized using iTOL (https://itol.embl.de/ accessed on 5 July 2026). ExPASy (https://web.expasy.org/compute_pi/ accessed on 5 July 2026) was used to analyze physicochemical properties of BrTGA proteins (number of amino acid, molecular weight, pI, GRAVY), and their subcellular localization (Table 1) was predicted with Plant-mPLoc (http://www.csbio.sjtu.edu.cn/bioinf/plant-multi/ accessed on 5 July 2026). Ka and Ks values and their ratios (Ka/Ks) were calculated for the identified paralogous gene pairs using the Simple Ka/Ks Calculator (NG) in TBtools to assess evolutionary selection pressure. To explore the evolutionary relationships between TGA genes of RCBr and other species, synteny analyses were performed with *B. rapa* cv. Chiifu, *B. napus* L., *S. lycopersicum*, *G. max* and *O. sativa*. The synteny analysis was conducted using the One Step MCScanX program embedded in TBtools, and the results were visualized with TBtools. Genomic data of *B. rapa* cv. Chiifu, *B. napus* L., *G. max*, *S. lycopersicum*, and *O. sativa* were retrieved from public databases including Ensembl Plants (https://plants.ensembl.org/) (accessed on 9 July 2026) and BnIR (http://yanglab.hzau.edu.cn/BnIR/ accessed on 9 July 2026).

### 2.4. Characterization of BrTGA Gene Family and Prediction of Promoter Cis-Acting Elements

Genomic data of RCBr were used to analyze conserved motifs of BrTGA proteins using TBtools (v2.487) (the maximum number of motifs was set to 8), and integrated visualization of the phylogenetic tree, conserved motifs, and domains was conducted [32]. The tertiary structures of BrTGA proteins were predicted using AlphaFold3 (https://alphafoldserver.com/ accessed on 27 September 2026) and visualized using the open-source PyMOL (v3.1.0). The 2500 bp promoter sequences upstream of *BrTGA* genes were extracted, and cis-acting elements within these sequences were predicted using the PlantCARE database.

### 2.5. Extraction of Total RNA and RT-qPCR Assays

Total RNA was extracted from roots, stems, cotyledons, true leaves, inflorescences, and siliques using a FastPure Plant Total RNA Isolation Kit (Vazyme Biotech, Nanjing, China), with a minimum of three biological replicates for each tissue. Total RNA was reverse-transcribed following the manufacturer’s protocol of the Reverse Transcription Kit (Accurate Biology, Cat. AG11705, Changsha, China). RT-qPCR was performed to detect gene expression levels with SYBR^®^ Green PCR Master Mix (Accurate Biology, Cat. AG11701, Changsha, China). The amplification profile was 95 °C for 30 s, followed by 40 cycles at 95 °C for 5 s, 60 °C for 30 s. Upon completion of the reaction, amplification curves and melting curves were checked (Figures S1 and S2). *BrCYP* was used as the reference gene to normalize gene expression levels among all samples [33,34]. Relative expression levels were calculated based on the 2^−ΔΔCt^ method [35]. All primer sequences are listed in Table S2, and the amplification efficiency and R^2^ values of all primers are provided in Table S3.

## 3. Results

### 3.1. Identification and Analysis of TGA Gene Family Members

**Figure 1:**
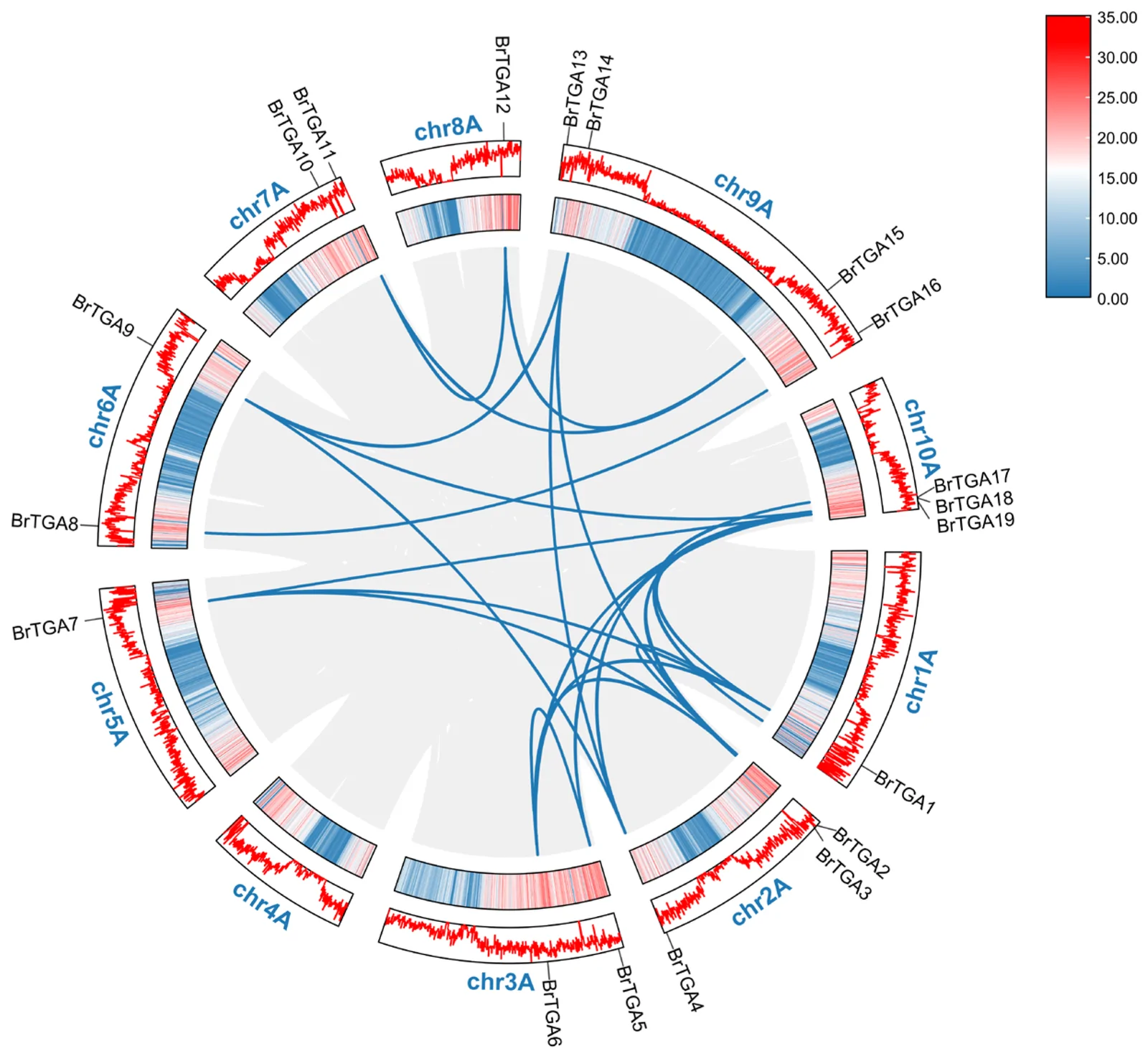
Chromosomal distribution and synteny analysis of *BrTGA* genes in RCBr. The color scale from blue to red represents gene density (genes per Mb). The blue lines in the inner circle represent collinear gene pairs of *BrTGA* genes.

Candidate TGA genes in RCBr were identified through BLAST and HMM searches, followed by CDD and Pfam domain verification and phylogenetic analysis. Sequences clustered in clade D were retained, resulting in 19 TGA genes identified in RCBr (Table S4). These genes are distributed across 9 chromosomes (Figure 1). The predicted protein lengths ranged from 295 to 493 amino acid residues with molecular weights of 34.21–55.07 kDa. The isoelectric point (pI) of the proteins varied from 5.67 to 9.06, and the grand average of hydropathicity (GRAVY) values ranged from −0.716 to −0.381, indicating that all 19 members are hydrophilic proteins. Subcellular localization prediction revealed that BrTGA proteins are localized in the nucleus.

To explore the evolutionary origin of the *BrTGA* gene family, we performed intra-genomic synteny analysis. The results revealed extensive segmental duplications across the RCBr genome, a hallmark of conserved genomic structure arising from the ancestral whole-genome triplication (WGT) event. Among the identified family members, *BrTGA18* and *BrTGA19* were tandemly duplicated genes with adjacent physical positions on chromosome 10, indicating that they originated from local tandem duplication after species divergence rather than from the ancient WGT event. The remaining collinear TGA gene pairs showed inter-chromosomal collinearity, demonstrating that the expansion of the *BrTGA* gene family is mainly driven by large-scale segmental duplications derived from WGT. No extensive loss of homologous gene copies was evident during evolution. Furthermore, the Nei–Gojobori (NG) algorithm was applied to assess the evolutionary constraints on 21 paralogous gene pairs screened from the *BrTGA* family. All valid gene pairs exhibited Ka/Ks ratios less than 1, indicating that the TGA gene family has been subjected to strong, pervasive purifying selection over long-term evolution (Table S5).

**Table 1:** Identification of TGA genes in RCBr.

Gene Name	Number of Amino acids	Molecular Weight (kDa)	Isoelectric Point	Instability Index	Aliphatic Index	Grand Average of Hydropathicity	Subcellular Location
*BrTGA1*	335	37.25	7.89	50.18	81.07	−0.533	Nucleus
*BrTGA2*	329	36.98	9.06	60.19	74.22	−0.716	Nucleus
*BrTGA3*	362	41.59	7.22	42.73	76.82	−0.531	Nucleus
*BrTGA4*	370	42.24	8.32	38.97	77.00	−0.537	Nucleus
*BrTGA5*	454	50.55	6.60	62.10	76.37	−0.579	Nucleus
*BrTGA6*	331	36.83	8.99	54.67	76.16	−0.606	Nucleus
*BrTGA7*	331	36.85	8.66	52.03	79.43	−0.597	Nucleus
*BrTGA8*	479	53.37	7.28	60.82	74.80	−0.556	Nucleus
*BrTGA9*	367	41.95	7.30	41.11	75.23	−0.545	Nucleus
*BrTGA10*	442	49.78	5.72	47.93	77.94	−0.484	Nucleus
*BrTGA11*	374	42.89	5.81	65.73	84.28	−0.599	Nucleus
*BrTGA12*	314	35.32	5.67	56.60	70.22	−0.686	Nucleus
*BrTGA13*	295	34.21	8.46	36.09	84.95	−0.381	Nucleus
*BrTGA14*	390	44.55	8.28	44.71	78.79	−0.546	Nucleus
*BrTGA15*	380	43.05	5.90	58.41	76.32	−0.640	Nucleus
*BrTGA16*	493	55.07	7.04	57.49	75.46	−0.528	Nucleus
*BrTGA17*	388	44.72	6.47	42.06	81.21	−0.443	Nucleus
*BrTGA18*	327	36.36	8.92	56.70	83.64	−0.493	Nucleus
*BrTGA19*	450	50.07	6.74	62.27	77.89	−0.551	Nucleus

### 3.2. Collinearity and Homologous Evolution of BrTGA Genes

**Figure 2:**
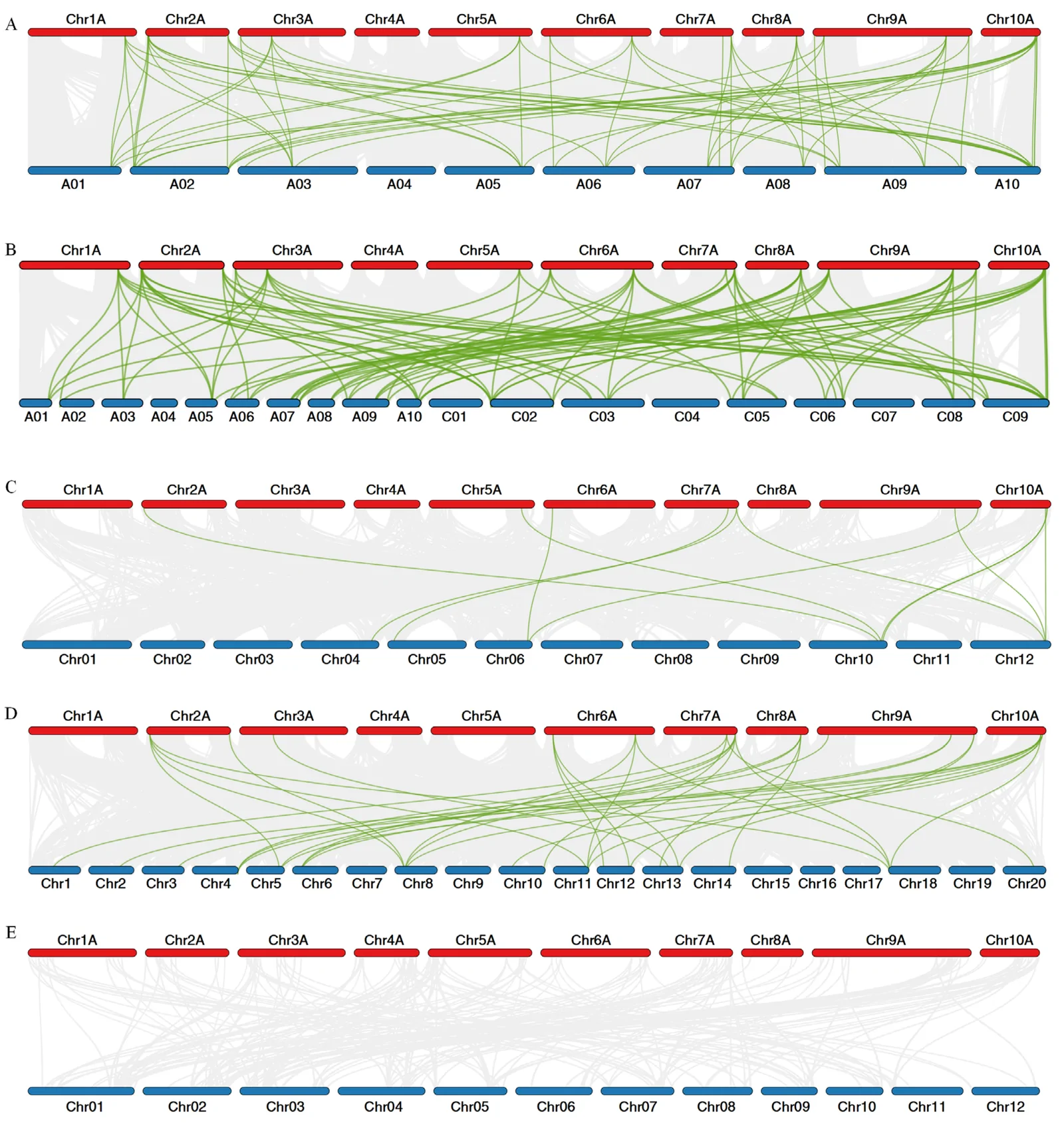
Collinearity analysis of the TGA gene family between RCBr and five representative plant species. (**A**) *B. rapa* cv. Chiifu; (**B**) *B. napus*; (**C**) *S. lycopersicum*; (**D**) *G. max*; (**E**) *O. sativa*. Gray lines in the background indicate genomic collinear regions among different species, and the highlighted green lines represent collinear orthologous pairs of TGA genes between RCBr and the five plant species.

To further dissect the phylogenetic evolutionary mechanism of the *BrTGA* gene family from RCBr, five species were selected for collinearity analysis (Figure 2). The five comparison species consisted of two Brassicaceae taxa: *B. rapa* cv. Chiifu (*B. rapa*, the subspecies genetically closest to RCBr) and *B. napus* L., plus three outgroup species (tomato, soybean, and rice). Collinearity analysis revealed that the counts of collinear homologs of *BrTGA* genes differed greatly across species, showing a general evolutionary pattern that closely related taxa retained higher collinear conservation.

Among all comparisons, RCBr *BrTGA* genes generated the largest number of collinear pairs with *B. napus* (91 pairs), followed by Chiifu (64 pairs), soybean (36 pairs), and tomato (11 pairs), while no collinear homologs were detected in rice. *B. napus* is an allotetraploid composed of A and C subgenomes, with its A subgenome derived from ancestral *B. rapa*. Both the A and C subgenomes retain abundant conserved collinear fragments, which can form collinear pairs with RCBr *BrTGA* homologs together, so more collinear homologs were identified in *B. napus* than in diploid Chiifu.

Notably, among the five species subjected to collinearity analysis, collinear orthologs of *BrTGA14* from RCBr were only detected in *B. rapa* cv. Chiifu. A large number of collinear homologs were identified across Brassicaceae, implying that the *BrTGA* gene family was highly conserved in the common ancestor of Brassicaceae. We observed generally higher collinear homology within dicots, whereas no collinear homologs were found in rice. This observation suggests extensive sequence divergence or gene loss of TGA genes in monocot lineages. In addition, relatively abundant collinear homologs were detected in soybean, further supporting the general conservation of the TGA gene family across dicot plants.

### 3.3. Phylogenetic Analysis of BrTGA Genes

**Figure 3:**
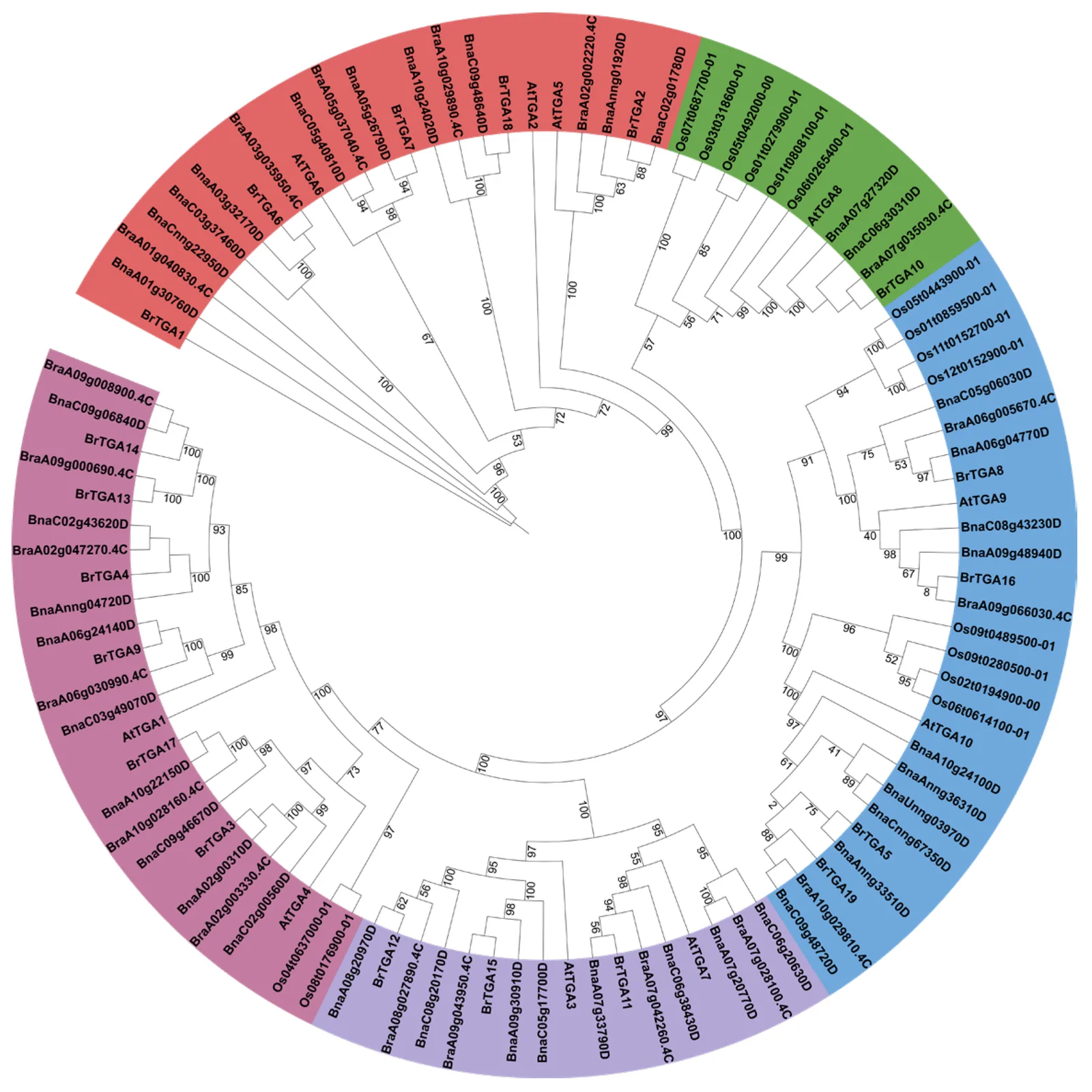
Maximum likelihood (ML) phylogenetic tree of TGA proteins. The tree was built with 5000 bootstrap replicates, and five clades were defined based on the phylogenetic relationships of *A. thaliana* TGA proteins.

To elucidate the evolutionary relationships among BrTGA proteins, homologous TGA sequences from the selected species were retrieved to construct a phylogenetic tree using the One-step ML Tree function in TBtools, including 10 from *A. thaliana*, 16 from *O. sativa*, 19 from *B. rapa* cv. *Chiifu*, 39 from *B. napus* L., and 19 from RCBr. According to the subfamily classification criteria of TGA transcription factors in *A. thaliana*, these sequences were divided into five clades. Specifically, *BrTGA3*, *BrTGA4*, *BrTGA9*, *BrTGA13*, *BrTGA14*, and *BrTGA17* were clustered into Group I together with *AtTGA1* and *AtTGA4*; *BrTGA1*, *BrTGA2*, *BrTGA6*, *BrTGA7*, and *BrTGA18* belonged to Group II with *AtTGA2*, *AtTGA5*, and *AtTGA6*; *BrTGA11*, *BrTGA12*, and *BrTGA15* were assigned to Group III along with *AtTGA3* and *AtTGA7*; *BrTGA5*, *BrTGA8*, *BrTGA16*, and *BrTGA19* fell into Group IV with *AtTGA9* and *AtTGA10*; and *BrTGA10* was grouped into Group V with *AtTGA8*. No species-specific clades were identified, suggesting high conservation of TGA homologs within Brassicaceae (Figure 3).

### 3.4. Conserved Motifs, Gene Structures, and Predicted Protein Structures of the BrTGA Family

**Figure 4:**
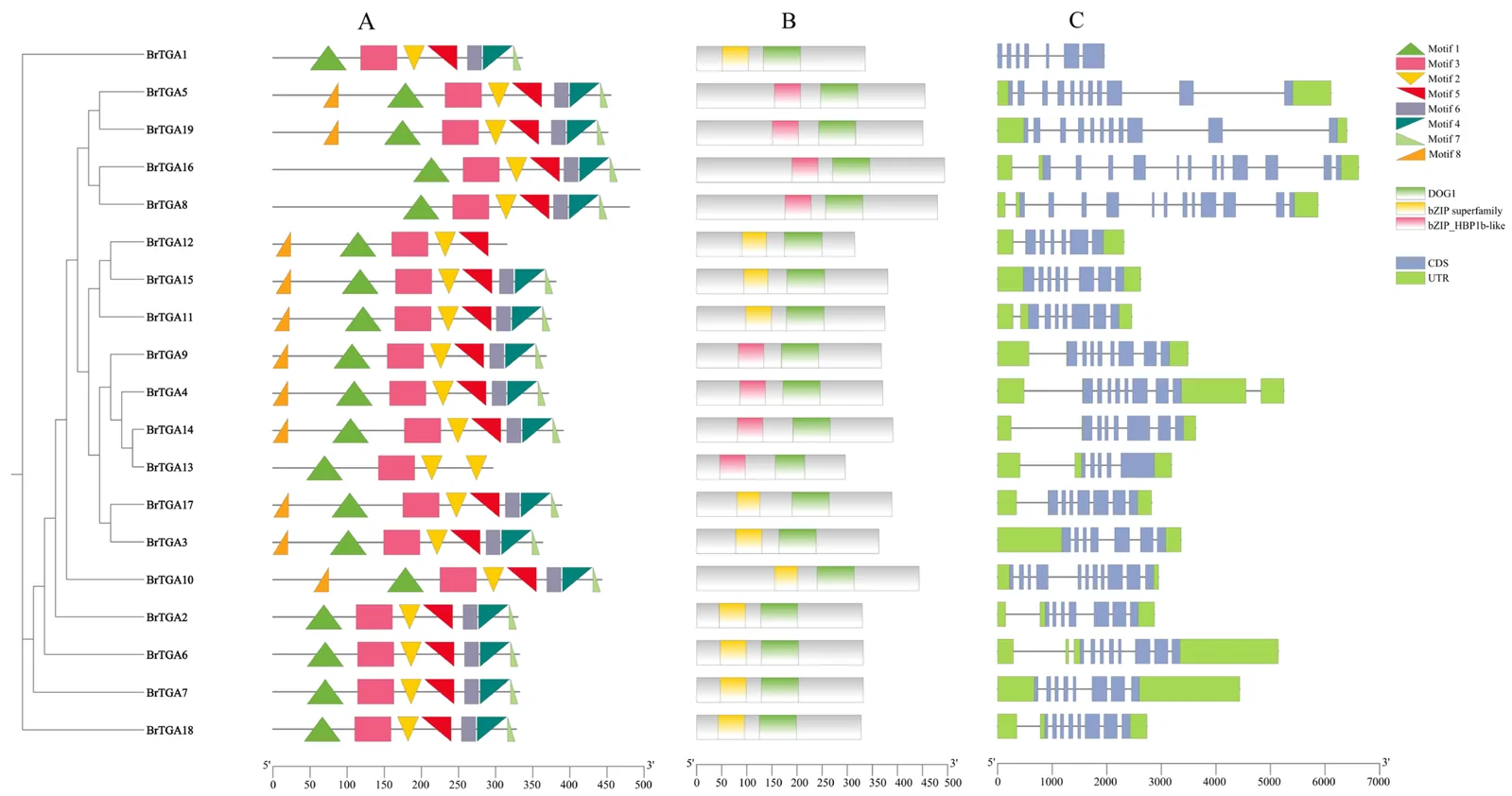
Phylogenetic connections, conserved motifs, protein domains, and gene structures of *BrTGA*. (**A**), conserved motifs of BrTGA. (**B**), domains of BrTGA. (**C**), gene structures of *BrTGA*.

Conserved motif analysis of the 19 BrTGA proteins identified eight conserved motifs (Motif 1–Motif 8) (Figure 4A). Among them, Motifs 1, 2, 3 were present in all BrTGA proteins. Notably, BrTGA13 contained only four conserved motifs, including two copies of motif 2, suggesting a relatively simple motif composition and potential structural divergence. The sequence logo of the eight conserved motifs (Figure S3) illustrates the degree of conservation of amino acid residues at each position. Gene structure analysis revealed that *BrTGA* genes contained 4–11 introns and 5–12 exons, indicating a certain degree of structural divergence among *BrTGA* genes during evolution (Figure 4C).

**Figure 5:**
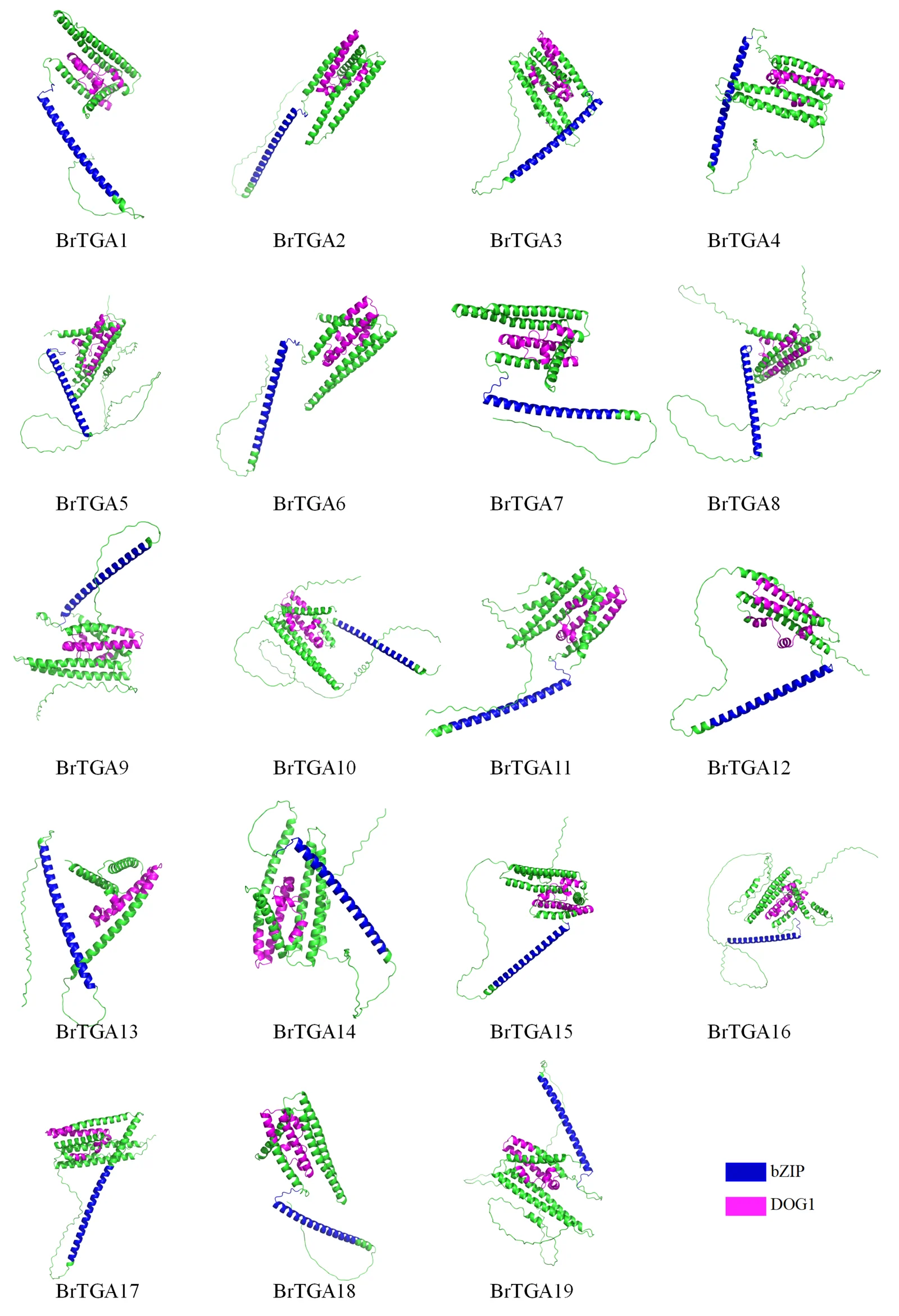
Predicted three-dimensional structures and structural characteristics of BrTGA proteins. The blue squares represent the bZIP domain, and magenta squares represent the DOG1 domain.

Conserved domain analysis showed that all 19 BrTGA proteins contained the characteristic bZIP and DOG1 domains (Figure 4B, Table S6). Furthermore, predicted three-dimensional structures showed that BrTGA proteins generally exhibited similar structural characteristics, particularly in regions corresponding to the conserved domains, although certain structural differences were observed among some family members (Figure 5). Taken together, the BrTGA family exhibited both a high degree of structural conservation and a certain degree of structural divergence at the levels of gene structure, conserved motifs, conserved domains, and predicted protein structures.

### 3.5. Cis-Acting Element Analysis of BrTGA Promoters

**Figure 6:**
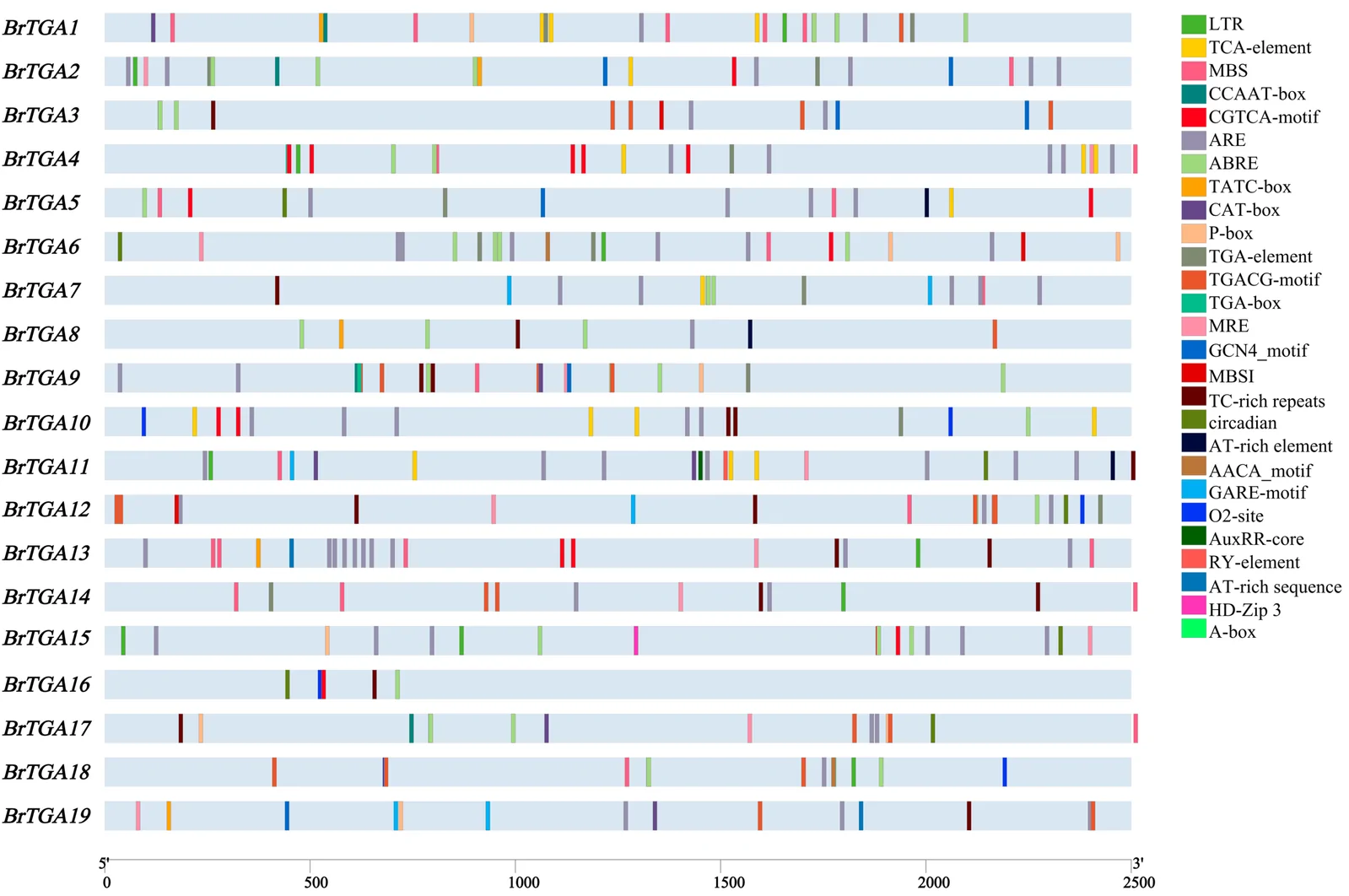
Predicted cis-elements in *BrTGA* promoters. Promoter sequences (2500 bp upstream of the start codon) of *BrTGA* genes were analyzed using the PlantCARE online database.

Cis-acting element prediction was performed on promoter sequences of the 19 *BrTGA* family members to explore their regulatory characteristics and biological functions. Three categories of functional cis-elements, including stress-responsive, hormone-responsive, and growth/development-related elements, were widely distributed across these promoters (Figure 6).

Stress-related cis-motifs included the anaerobic induction motif ARE, drought-responsive MBS, low-temperature-responsive LTR, and defense-associated TC-rich repeats. Hormone-responsive cis-elements contained motifs responding to abscisic acid, methyl jasmonate, salicylic acid, auxin and gibberellin. In addition, several cis-elements involved in meristem proliferation, circadian rhythm, endosperm and seed development were detected, suggesting potential regulatory roles of *BrTGA* genes in plant growth and organ morphogenesis.

Collectively, cis-element distribution analysis indicated that the promoters of *BrTGA* family genes harbor abundant development-associated cis-elements, together with hormone and environmental responsive motifs, implying that this gene family participates in the growth and development of *B. rapa*.

### 3.6. BrTGA Expression in Different Tissues

**Figure 7:**
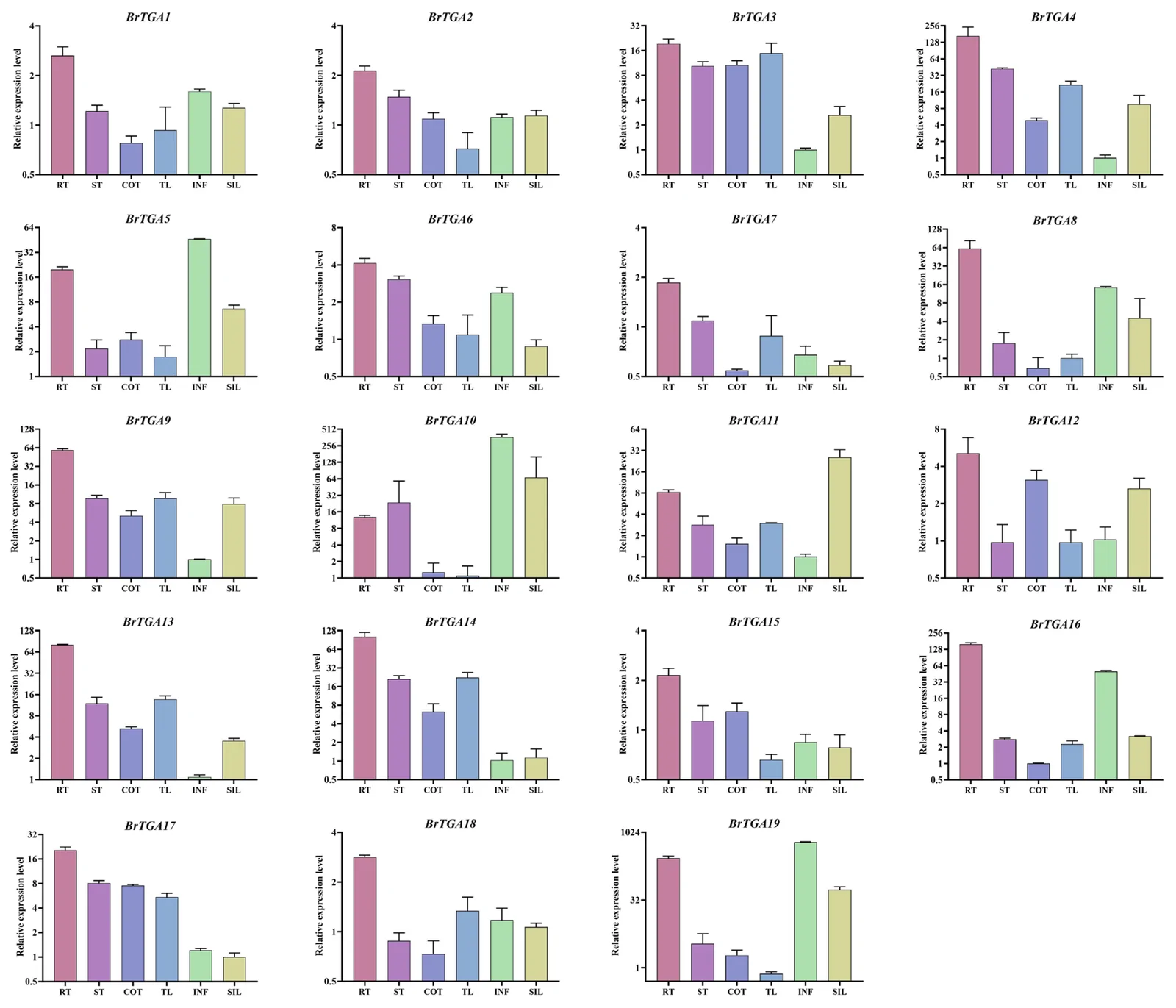
Expression profiles of *BrTGA* genes in various tissues. RT, root; ST, stem; COT, cotyledon; TL, true leaf; INF, inflorescence; SIL, silique. The y-axis represents relative expression level plotted on a log_2_ scale. Error bars represent the standard deviations (*n* = 3).

The relative expression levels of 19 *BrTGA* genes were analyzed in six tissues, including roots, stems, cotyledons, true leaves, inflorescences, and siliques (Figure 7). The results showed that the *BrTGA* family members exhibited distinct expression patterns across different tissues. Overall, most *BrTGA* genes showed relatively high expression levels in roots, suggesting that some *BrTGA* genes may play important roles in root development and related physiological processes.

Different subgroups and family members exhibited varying degrees of tissue-specific expression preferences. The *BrTGA1*, *BrTGA2*, *BrTGA6*, *BrTGA7*, and *BrTGA18* genes in Group II generally showed preferential expression in roots, although differences in transcript abundance were observed among individual members in cotyledons, true leaves, and siliques, suggesting potential divergence in tissue-specific expression patterns among members of the same subgroup. *BrTGA10* in Group V exhibited a pronounced preference for expression in inflorescences, whereas its expression level was lowest in true leaves. In Group IV, *BrTGA5*, *BrTGA8*, *BrTGA16*, and *BrTGA19* showed relatively high expression levels in roots, inflorescences, and siliques, indicating a relatively broad tissue expression pattern.

In addition, several genes, including *BrTGA3*, *BrTGA4*, *BrTGA9*, *BrTGA11*, *BrTGA13*, and *BrTGA14*, exhibited relatively low expression levels in inflorescences. The expression levels of different *BrTGA* genes also varied considerably among stems, cotyledons, and siliques. Overall, the 19 *BrTGA* genes displayed diverse tissue expression patterns, characterized by both shared expression trends among family members and distinct tissue preferences and expression divergence. These differential expression patterns suggest that *BrTGA* family members may perform distinct functions in the growth, development, and physiological processes of different tissues in RCBr.

## 4. Discussion

TGA transcription factors constitute an important subgroup of the plant bZIP superfamily and function as key regulators integrating phytohormone signaling, abiotic stress tolerance, and plant developmental processes [4]. To date, genome-wide identification and functional characterization of the TGA gene family have been conducted in multiple plant species. Ten TGA members have been identified in *A. thaliana* [7], 7 in sweet orange [36], 27 in soybean [37], 20 in peanut [38], 38 in wheat [9], and 39 in *B. napus* L. [11].

The ancient whole-genome triplication (WGT) event in the Brassica lineage provided an important basis for gene family expansion, while subsequent gene fractionation and gene loss further shaped the present-day composition of gene families [39,40]. In this study, 19 *BrTGA* genes were identified in both RCBr and the *B. rapa* cv. Chiifu, indicating that the TGA gene family is relatively conserved in size among different *B. rapa* germplasms. This relatively stable family size may reflect a degree of selective constraint on *BrTGA* genes during the evolution of the *B. rapa* genome, contributing to the maintenance of a relatively stable gene complement over evolutionary time.

The *BrTGA* family exhibits both high sequence conservation and pronounced functional diversification. All *BrTGA* proteins contain three core conserved motifs as well as intact bZIP and DOG1 functional domains. The bZIP domain is a signature domain of TGA transcription factors and is involved in DNA binding and protein dimerization. The DOG1 domain was initially identified in proteins involved in the regulation of seed dormancy in Arabidopsis. Its widespread presence in the *BrTGA* family suggests that, in addition to their basic transcriptional regulatory functions, BrTGA proteins may also participate in the regulation of multiple developmental pathways, including seed dormancy and germination, providing clues for further functional validation.

These structural features are highly conserved among TGA proteins from sweet orange, peanut, and *B. napus* L., suggesting that they represent common structural characteristics of plant TGA proteins [11,36,38]. Differences in N-terminal motif composition and exon–intron organization among subfamilies suggest that *BrTGA* paralogs have undergone a certain degree of structural divergence during evolution, which may have contributed to the development of functional specificity [4,41].

Promoter cis-acting element analysis revealed that the promoter regions of *BrTGA* genes contained various hormone-responsive elements, including salicylic acid (SA), jasmonic acid (JA), abscisic acid (ABA), and gibberellin (GA) response elements, as well as multiple stress-responsive regulatory elements, suggesting that *BrTGA* genes may be co-regulated by multiple phytohormone signaling pathways and environmental stresses. Previous studies have shown that TGA transcription factors participate in SA-mediated immune responses and exhibit complex interactions with JA/ethylene signaling and other hormone pathways [42,43,44]. In addition, some TGA members have been shown to participate in responses to abiotic stresses, such as drought and salt stress, and may be associated with the ABA signaling pathway [37]. Therefore, *BrTGA* genes may be involved in the growth and development and stress responses of RCBr, although their specific biological functions require further validation through molecular and genetic approaches. It should be noted that the present study focuses on revealing the molecular features of the *BrTGA* gene family in RCBr. Therefore, cis-element analysis was performed only on *BrTGA* promoters. Comparative analysis incorporating homologous genes from multiple species including *A. thaliana* could better clarify the similarities and differences in cis-regulatory patterns of the TGA gene family across species and provide a more comprehensive perspective for understanding the evolution of TGA regulatory characteristics.

Tissue expression profiling further revealed differential expression patterns and potential functional partitioning of *BrTGA* genes across different tissues of RCBr. Analysis of transcript abundance across multiple tissues showed that *BrTGA* genes exhibited distinct tissue-specific and, to some extent, subfamily-specific expression patterns. Overall, most *BrTGA* genes showed relatively high expression levels in roots, suggesting that some *BrTGA* members may play important roles in root development and in the response of roots to external signals and stresses. Similar root-preferential expression patterns have also been reported for TGA genes in common bean [8], suggesting that TGA family members may have conserved functions in root development and responses to belowground environmental signals.

## 5. Conclusions

In summary, this study systematically characterized the *BrTGA* gene family in RCBr, revealing its evolutionary relationships, gene and protein structural features, conserved domains and motifs, promoter cis-acting elements, and tissue-specific expression patterns. These findings provide a comprehensive overview of the molecular characteristics and potential regulatory roles of *BrTGA* genes and provide a basis for further functional investigation of their roles in growth, development, and stress responses in *B. rapa*.

## Data Availability

The datasets used and/or analyzed during the current study were available from the corresponding author on reasonable request.

## Supplementary Materials

The following supporting information can be downloaded at: https://www.mdpi.com/article/10.3390/cimb48101089/s1.
