# Supplementary material for: Genome-Wide Identification and Expression Profiling of the TGA Gene Family in Rapid-Cycling Brassica rapa
Source: Curr Issues Mol Biol. 2026 Oct 21;48(10):1089. doi: 10.3390/cimb48101089 (PMC13703585; doi:10.3390/cimb48101089)
Supplement: Supplementary file 1 [file cimb-48-01089-s001.zip › Figure S1.pdf]

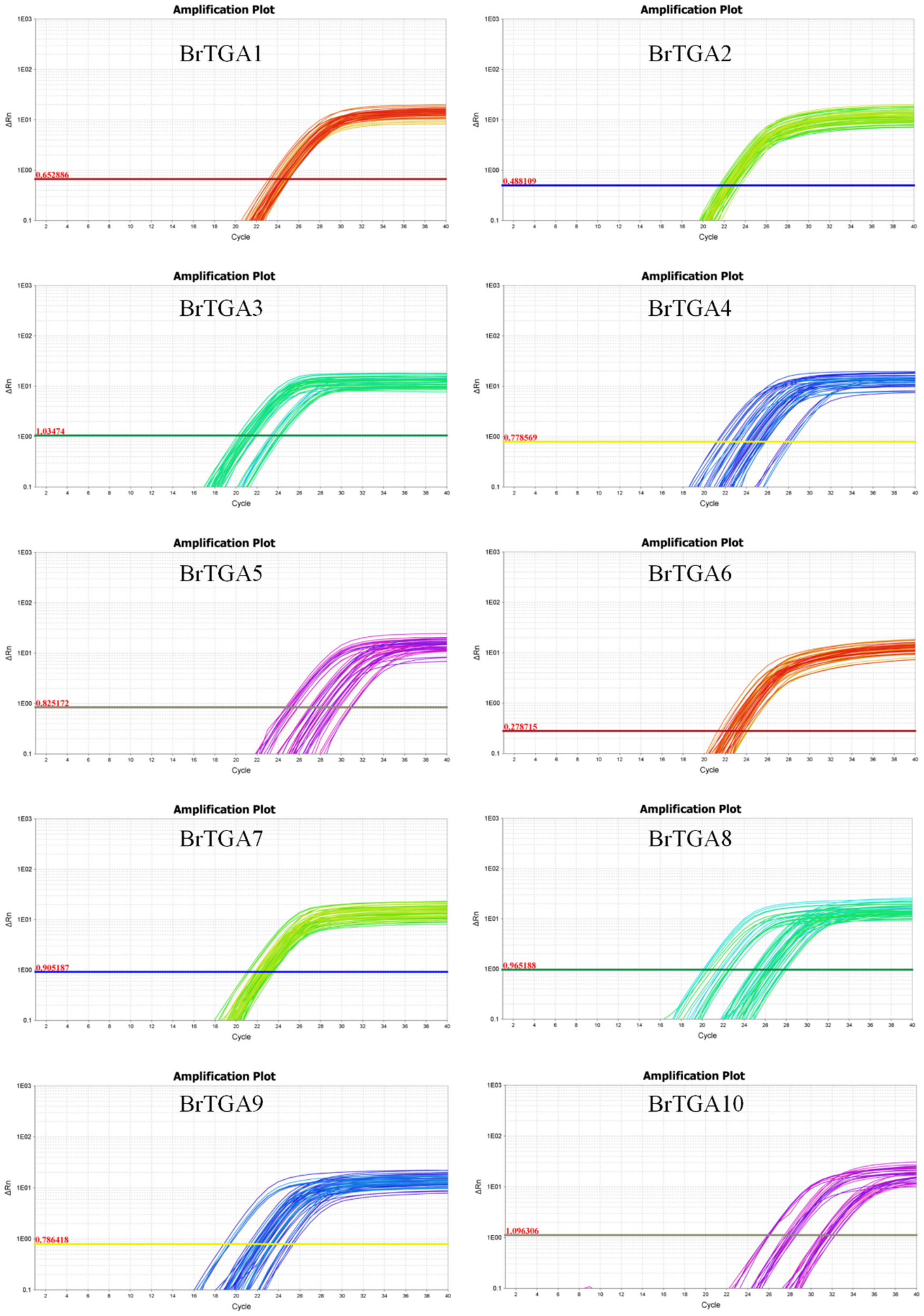
Amplification Plot
BrTGA1
0.652886
Amplification Plot
BrTGA2
0.488109
Amplification Plot
BrTGA3
1.03474
Amplification Plot
BrTGA4
0.778569
Amplification Plot
BrTGA5
0.825172
Amplification Plot
BrTGA6
0.278715
Amplification Plot
BrTGA7
0.905187
Amplification Plot
BrTGA8
0.965188
Amplification Plot
BrTGA9
0.786418
Amplification Plot
BrTGA10
1.096306
ΔRn
Cycle

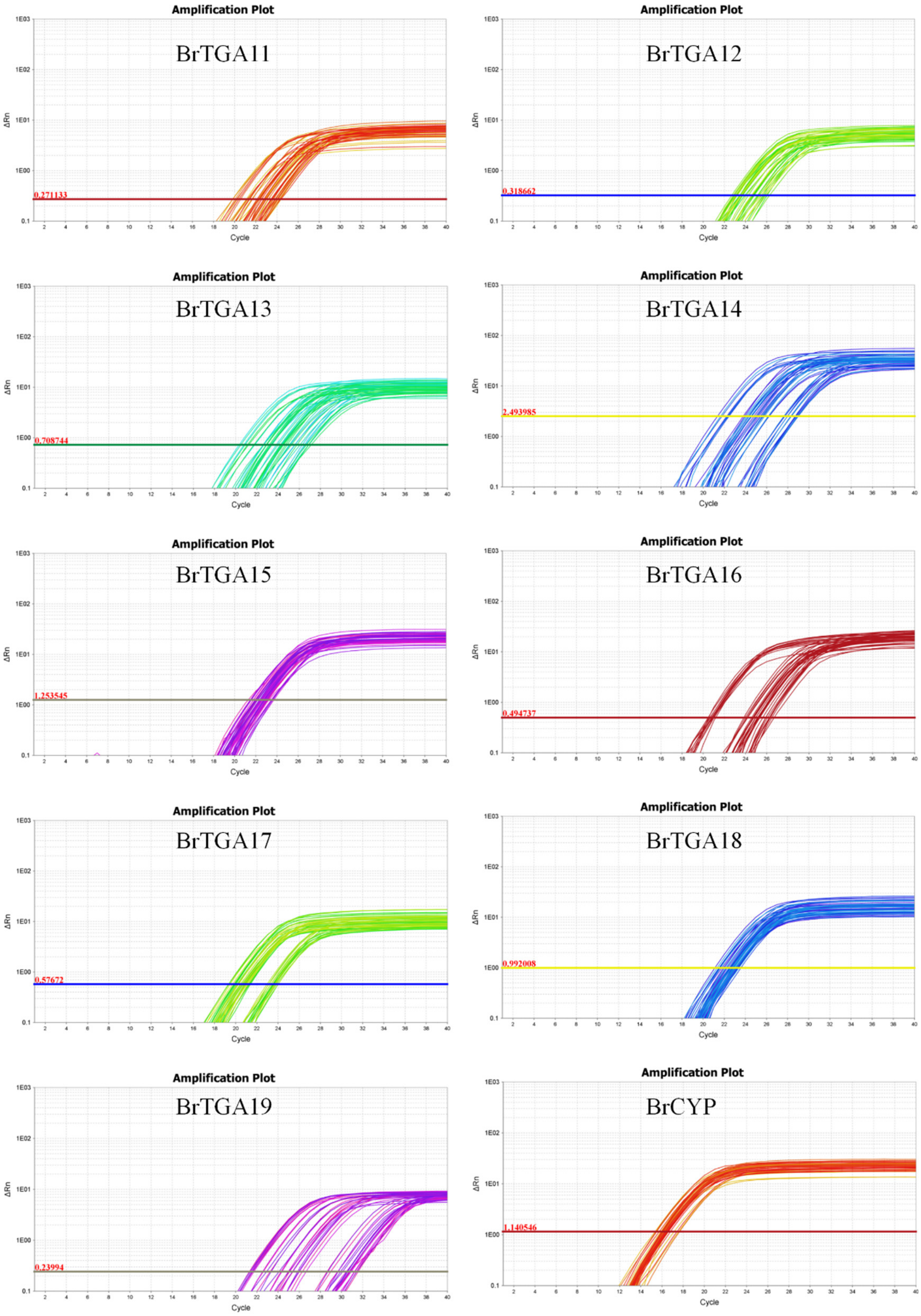

Figure S1. Amplification curves of fluorescence quantitative primers for TGA gene family members in RCBr.
