# Supplementary material for: Genome-Wide Identification and Expression Profiling of the TGA Gene Family in Rapid-Cycling Brassica rapa
Source: Curr Issues Mol Biol. 2026 Oct 21;48(10):1089. doi: 10.3390/cimb48101089 (PMC13703585; doi:10.3390/cimb48101089)
Supplement: Supplementary file 1 [file cimb-48-01089-s001.zip › Figure S2.pdf]

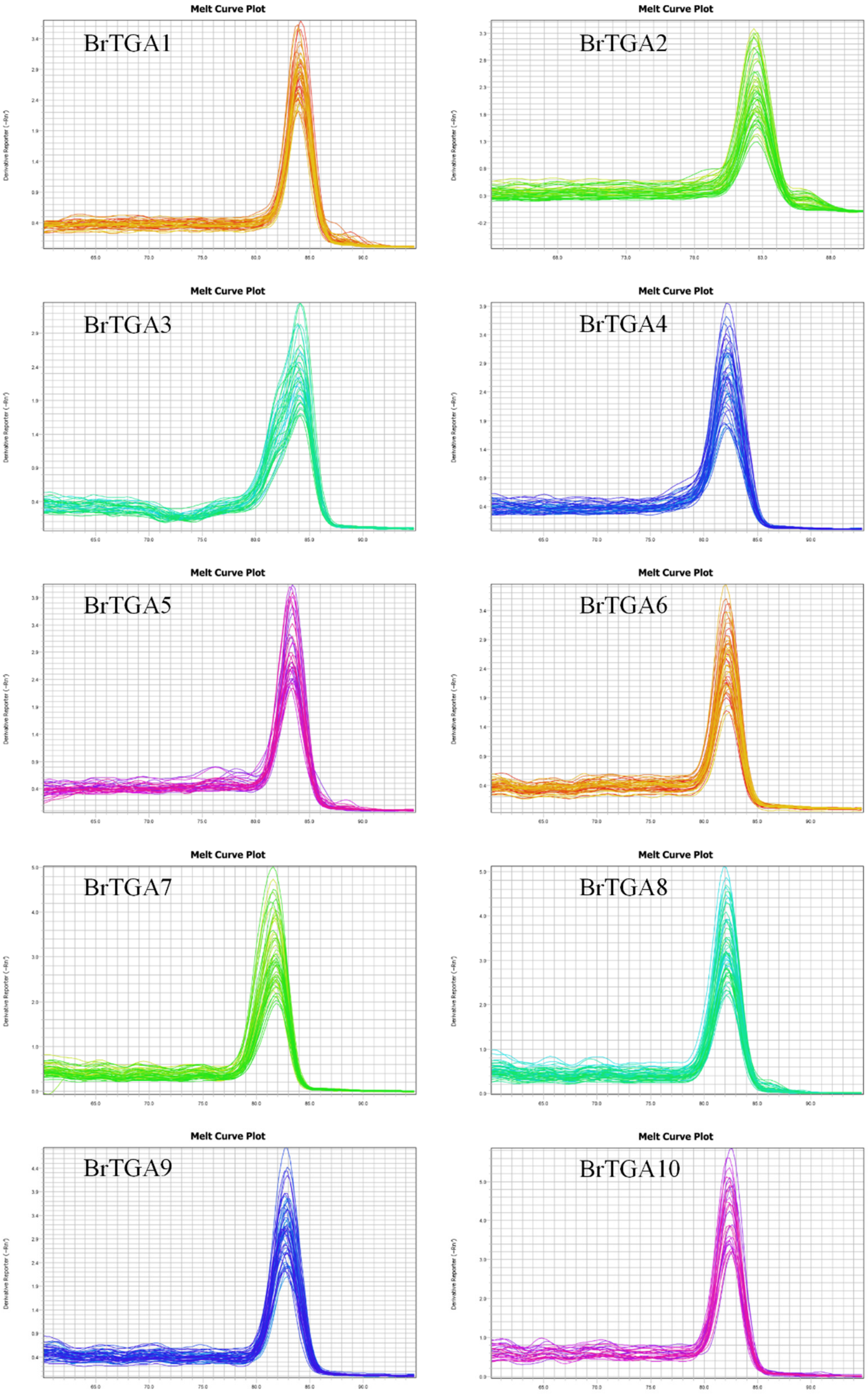
Melt Curve Plot
BrTGA1
Melt Curve Plot
BrTGA2
Melt Curve Plot
BrTGA3
Melt Curve Plot
BrTGA4
Melt Curve Plot
BrTGA5
Melt Curve Plot
BrTGA6
Melt Curve Plot
BrTGA7
Melt Curve Plot
BrTGA8
Melt Curve Plot
BrTGA9
Melt Curve Plot
BrTGA10

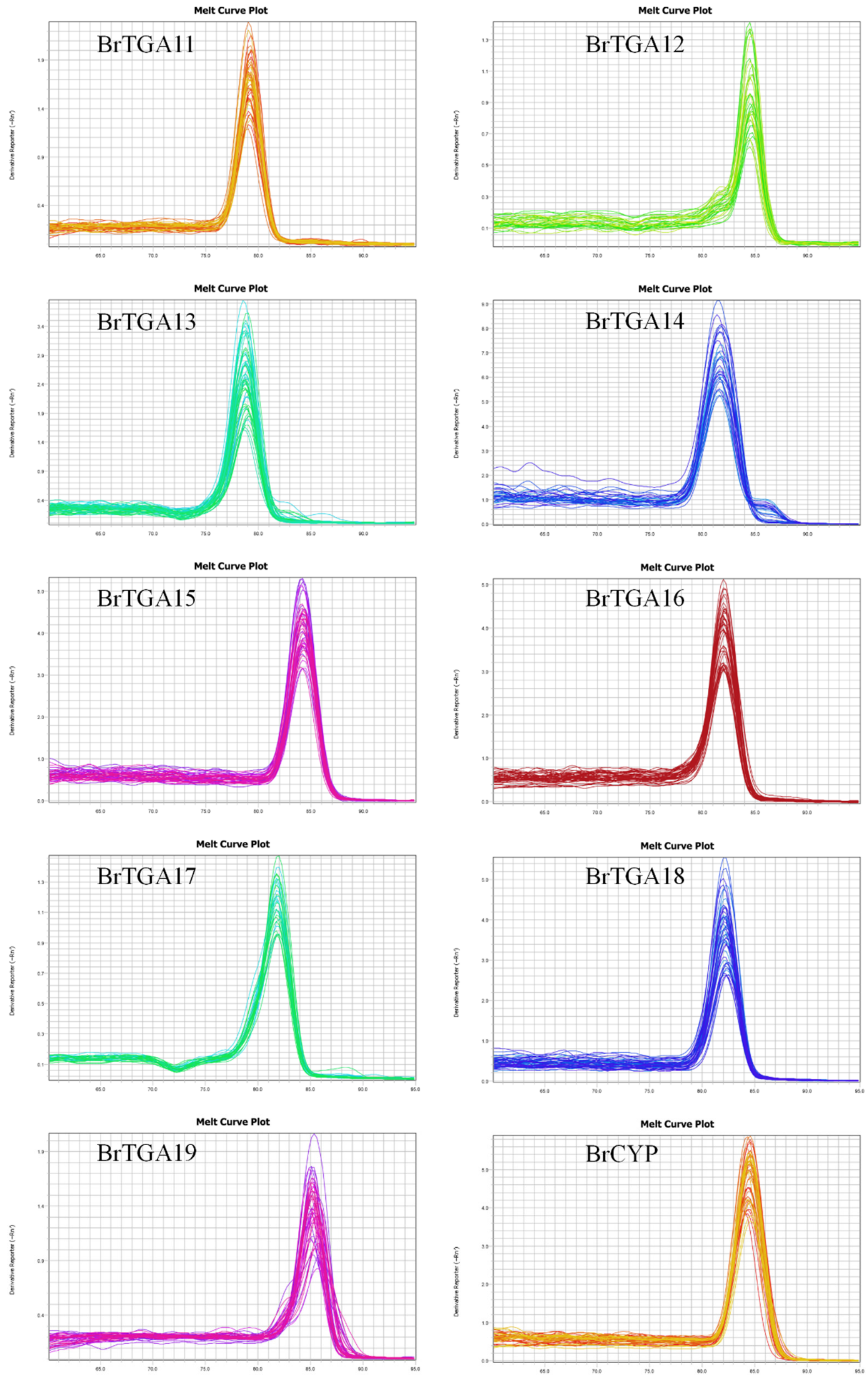

Figure S2. Melting curves of fluorescence quantitative primers for TGA gene family members in RCBr.
