# Supplementary material for: Genome-Wide Identification and Expression Profiling of the TGA Gene Family in Rapid-Cycling Brassica rapa
Source: Curr Issues Mol Biol. 2026 Oct 21;48(10):1089. doi: 10.3390/cimb48101089 (PMC13703585; doi:10.3390/cimb48101089)
Supplement: Supplementary file 1 [file cimb-48-01089-s001.zip › Figure S3.pdf]

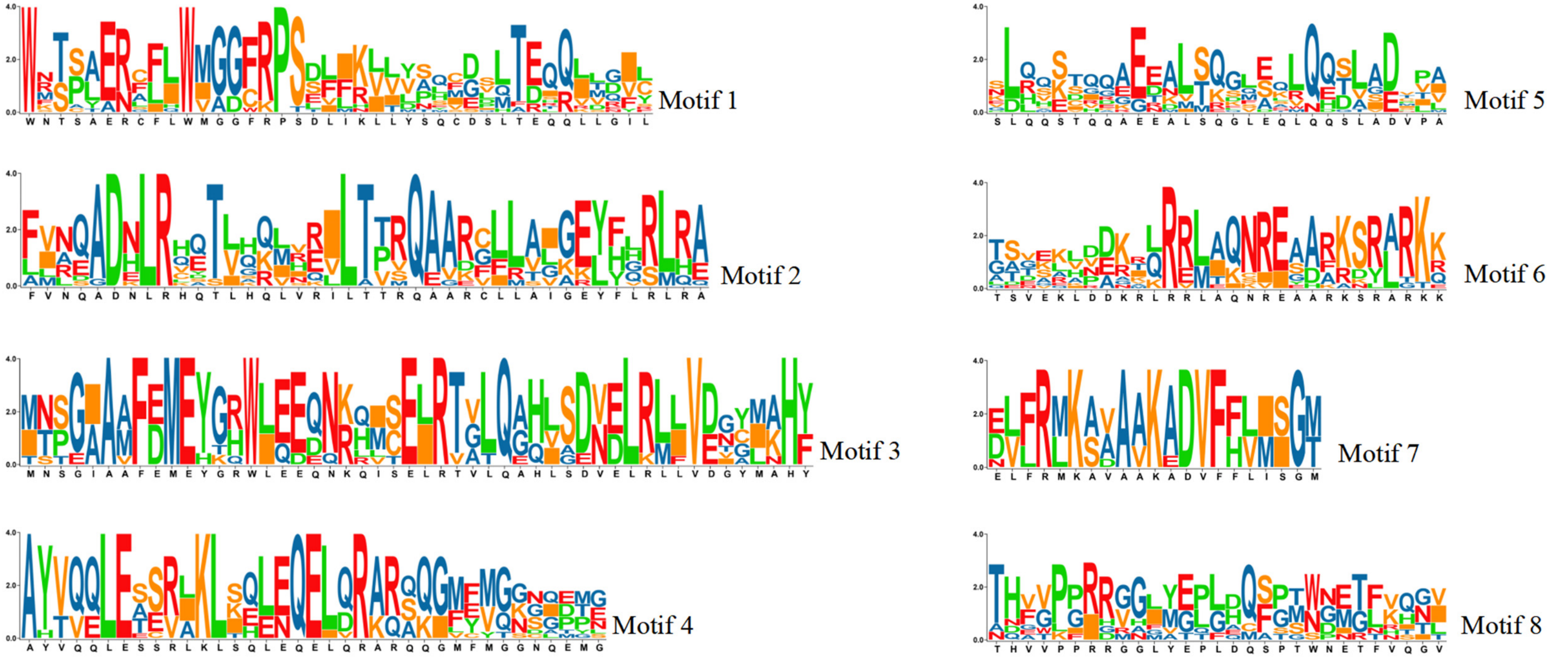

Figure S3. motif logos of the eight conserved motifs.
